# Impact of the 2025 New South Wales Respiratory Syncytial Virus Prevention Program on Infant Notifications and Hospitalisations: A Population‐Based Analysis

**DOI:** 10.5694/mja2.70222

**Published:** 2026-06-10

**Authors:** Janaki Amin, Sally L. Ellis, Christopher Lambeth, Jessica Gugusheff, Christine Selvey

**Affiliations:** ^1^ Health Protection NSW NSW Health St Leonards New South Wales Australia; ^2^ Department of Health Sciences and Nursing Macquarie University Sydney New South Wales Australia

**Keywords:** child health, maternal health, prevention and control, public health, respiratory tract infections, vaccination, vaccine preventable disease

## Abstract

The 2025 NSW RSV Prevention Program, which achieved an estimated coverage of 63% maternal vaccination and 18% for infant immunisation, led to more than 40% reduction in RSV notifications and hospitalisations among infants aged younger than 6 months.

## 1 Introduction

Respiratory syncytial virus (RSV) is a leading cause of infant morbidity [1]. Maternal RSV vaccination reduces severe RSV lower respiratory tract infections and hospitalisations in the first 3–6 months of life [2, 3]. In 2024, New South Wales implemented a targeted immunisation program with the monoclonal antibody Beyfortus (nirsevimab) for infants at high risk of severe outcomes. In 2025, this extended to a whole‐of‐population program with vaccination during pregnancy with RSV vaccine Abrysvo (recombinant respiratory syncytial virus pre‐fusion F protein), funded under the National Immunisation Program since February 2025, and administration of Beyfortus to infants whose mothers were not vaccinated or who were at risk of severe disease, and to medically high‐risk infants in their second year, funded by NSW Health [4]. While all Australian states and territories have implemented similar programs, this is one of the first reports on the population level impact of the 2025 NSW RSV Prevention Program.

## 2 Methods

Vaccinations with Abrysvo for females aged 14–54 years and doses of Beyfortus for infants aged < 18 months administered from 1 January to 31 December 2025 were extracted from the Australian Immunisation Register (AIR) on 4 March 2026. For the calendar years 2023 and 2025, NSW notification data for diagnosed RSV infections were extracted from the Notifiable Conditions Information Management System, and RSV hospitalisation data (diagnosis codes ICD‐10‐AM J12.1, J20.5, J21.0, B97.4) were extracted from the NSW Health Enterprise Data Warehouse. Projected single‐year population data from the Department of Planning, Housing and Infrastructure [5] were used to estimate the number of pregnancies and the infant population for calculation of immunisation coverage estimates and notification and hospitalisation rates, respectively. The number of pregnancies was estimated from the total fertility rate used in the projections and the average number of babies per pregnancy in NSW from 2023.

The rate ratios (RRs) for RSV notifications and hospitalisations were calculated for infants aged < 6 months compared to the reference age category 12–< 18 months, for 2023 and 2025. Comparison between age groups was used to account for differences in the amount of circulating RSV between years. The impact of the program was assessed by comparing each RR for 2023 with the respective RR for 2025. To indicate the precision of estimates, 95% confidence intervals are reported. All analyses were performed using R statistical software v4.4.1.

The *Public Health Act 2010* (NSW) [6] allows for release of data to identify and monitor risk factors for diseases and conditions that have a substantial adverse impact on the population and to improve service delivery. Monitoring effectiveness of vaccination programs is a defined purpose for use of AIR data in the *Australian Immunisation Register Act 2015* (Cth) [7]. The NSW Ministry of Health provided project oversight and approval for publication. This study is reported in accordance with Strengthening the Reporting of Observational Studies in Epidemiology guidelines [8] (Supporting Information).

## 3 Results

In 2025, 58,501 Abrysvo doses were recorded among an estimated 93,306 pregnancies—estimated coverage of 62.7%. The numbers of Beyfortus doses were 9626 for infants aged < 6 months and 266 for infants aged 12–< 18 months—estimated coverage of 18.5% and 0.5%, respectively.

**FIGURE 1:**
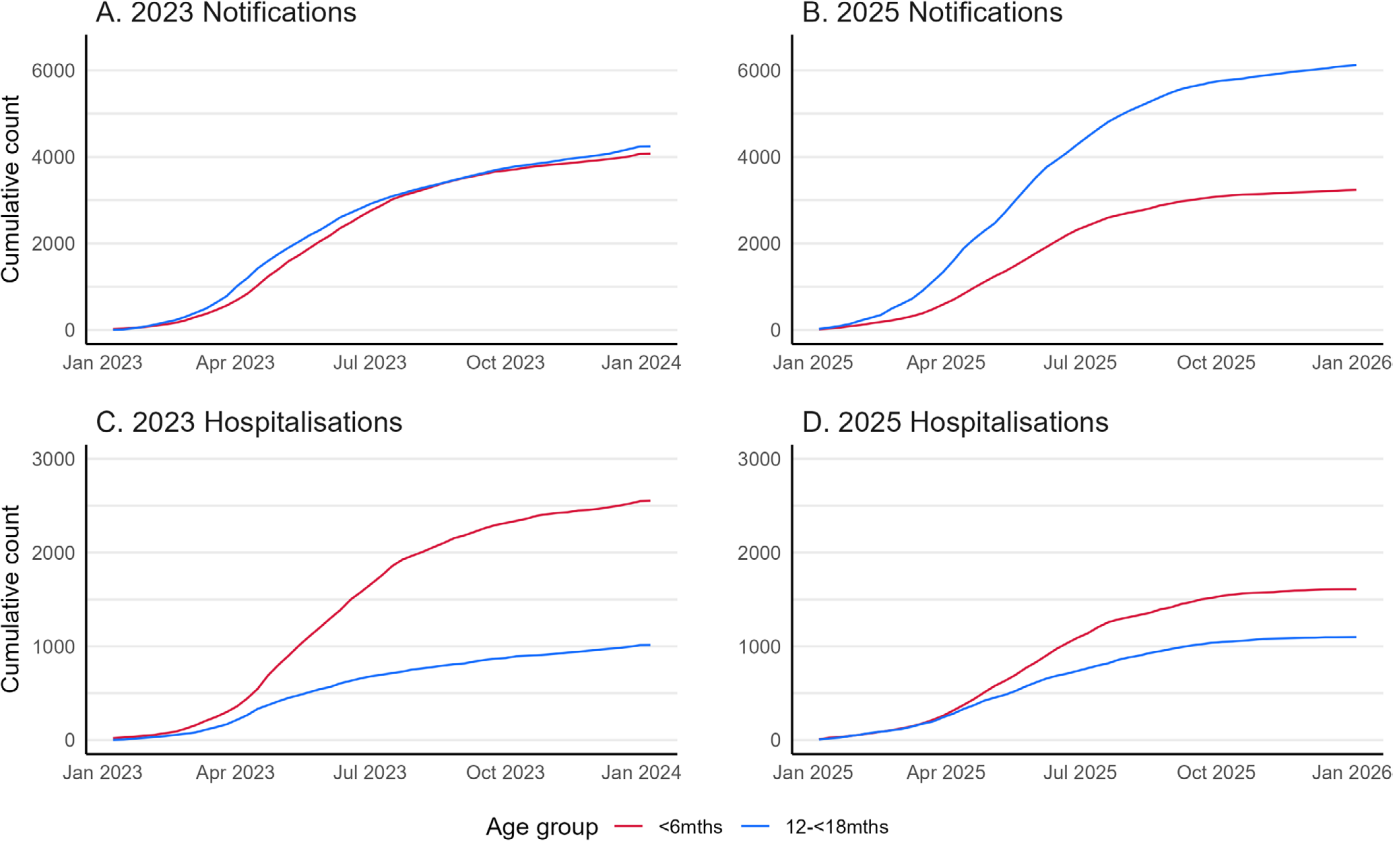
Cumulative counts of respiratory syncytial virus notifications and hospitalisations for infants < 6 months and 12 to < 18 months of age in 2023 (before the prevention program was implemented) and 2025 (after the prevention program was implemented).

RSV notifications increased from 46,484 in 2023 to 71,377 in 2025. In 2023, the numbers of notifications for infants aged < 6 months and 12–< 18 months were similar, while in 2025 notifications were markedly lower for infants aged < 6 months compared with those aged 12–< 18 months (Figure 1). While the annual notification rate for infants aged 12–< 18 months increased substantially between 2023 and 2025, it declined for infants aged < 6 months with a significant 46% reduction in the RR (Table 1).

**TABLE 1:** Numbers, rates and rate ratios of respiratory syncytial virus notifications and hospitalisations for infants < 6 months and 12–< 18 months of age in 2023 and 2025.

Year	Age group (months)	Number	Rate per 100,000 (95% CI)	Rate ratio (95% CI)
Notifications				
2023	0–< 6	4078	8587 (8336–8842)	0.96 (0.92–1.00)
	12–< 18	4246	8904 (8650–9163)	Ref
2025	0–< 6	3239	6210 (6004–6420)	0.52 (0.50–0.54)
	12–< 18	6122	12,040 (11,759–12,326)	Ref
Hospitalisations				
2023	0–< 6	2551	5371 (5170–5578)	2.53 (2.36–2.72)
	12–< 18	1013	2124 (1997–2258)	Ref
2025	0–< 6	1609	3085 (2938–3237)	1.43 (1.33–1.54)
	12–< 18	1099	2161 (2037–2292)	Ref.

In 2023, the number of hospitalisations for infants aged < 6 months was much greater than for those aged 12–< 18 months, while in 2025, hospitalisations for infants aged < 6 months approached those for infants aged 12–< 18 months (Figure 1, Table 1). The hospitalisation rates for infants aged 12–< 18 months were similar in 2023 and 2025, but for those aged 0–< 6 months the hospitalisation rate was substantially lower in 2025 than in 2023 and resulted in a significant 43% reduction in the RR.

## 4 Discussion

The absolute and relative declines in RSV notifications and hospitalisations among infants aged < 6 months from 2023 to 2025 demonstrates program impact in the age group most at risk of severe disease. Key limitations of this population‐based study are that it is ecological and cannot establish causality, and that only 1 year's data following implementation of the prevention program were available.

Despite these limitations, the consistency of the declines in notifications and hospitalisations in the target population, along with findings from clinical trials and emerging real‐world evidence [9, 10], support the likelihood of a true program effect. Continued national surveillance and planned linked data analyses will provide a more definitive assessment of the individual and population impact of the RSV prevention program on disease.

## Author Contributions

Conceptualisation: Janaki Amin (lead), Sally L. Ellis (equal), Jessica Gugusheff (equal), Christopher Lambeth (equal), Christine Selvey (equal). Data curation: Jessica Gugusheff (supporting), Christopher Lambeth (lead). Formal analysis: Christopher Lambeth (lead). Funding acquisition: Not applicable. Investigation: Sally L. Ellis (equal), Jessica Gugusheff (equal), Christopher Lambeth (equal). Methodology: Janaki Amin (equal), Sally L. Ellis (equal), Jessica Gugusheff (equal), Christopher Lambeth (equal). Project Administration: Janaki Amin (equal), Sally L. Ellis (equal), Christopher Lambeth (equal), Christine Selvey (equal). Resources: Sally L. Ellis (equal), Jessica Gugusheff (equal), Christopher Lambeth (equal). Software: Christopher Lambeth (lead). Supervision: Janaki Amin (lead). Validation: Jessica Gugusheff (Equal), Christopher Lambeth (equal). Visualisation: Christopher Lambeth (lead). Writing – original draft: Janaki Amin (lead), Sally L. Ellis (supporting), Jessica Gugusheff (supporting), Christopher Lambeth (supporting), Christine Selvey (supporting). Writing – review and editing: Janaki Amin (equal), Sally L. Ellis (equal), Jessica Gugusheff (equal), Christopher Lambeth (equal), Christine Selvey (equal).

## Funding

The authors have nothing to report.

## Disclosure

We used Microsoft Copilot (accessed March 2026) to assist with drafting and refining wording for sections of the manuscript. All content generated with assistance from Copilot was reviewed, edited and verified by the authors, who take full responsibility for the final manuscript. Not commissioned; externally peer‐reviewed.

## Conflicts of Interest

The authors declare no conflicts of interest.

## Supporting information


**Data S1:** STROBE Statement—Checklist of items that should be included in reports of cohort studies.

## Data Availability

De‐identified aggregate data can be made available on request pending approval of the relevant data custodian and stewards in accordance with applicable laws.
